# In Vitro Functional and Structural Evaluation of Low-Complexity Artificial Human Epidermis for 3D Tissue Engineering

**DOI:** 10.3390/bioengineering12030230

**Published:** 2025-02-24

**Authors:** Dorottya Kocsis, Dániel Sztankovics, Liza Józsa, Afrodité Németh, Tamás Garay, Márton Bese Naszlady, Miléna Lengyel, Miklós Vecsernyés, István Antal, Anna Sebestyén, Franciska Erdő

**Affiliations:** 1 Faculty of Information Technology and Bionics, Pázmány Péter Catholic University, Práter u. 50a., 1083 Budapest, Hungary; kocsis.dorottya@itk.ppke.hu (D.K.); afrodite.nemeth@gmail.com (A.N.); garay.tamas@itk.ppke.hu (T.G.); naszlady.marton.bese@itk.ppke.hu (M.B.N.); 2 Department of Pathology and Experimental Cancer Research, Semmelweis University, Üllői út 26., 1085 Budapest, Hungary; sztankovics.daniel@gmail.com (D.S.); hsebanna@gmail.com (A.S.); 3 Department of Pharmaceutical Technology, Faculty of Pharmacy, University of Debrecen, Nagyerdei krt. 98., 4032 Debrecen, Hungary; jozsa.liza@euipar.unideb.hu (L.J.); vecsernyes.miklos@pharm.unideb.hu (M.V.); 4 Department of Internal Medicine and Oncology, Semmelweis University, Korányi Sándor u. 2/a, 1083 Budapest, Hungary; 5 Department of Pharmaceutics, Semmelweis University, Hőgyes Endre u. 7, 1092 Budapest, Hungary; lengyel.milena@semmelweis.hu (M.L.); antal.istvan@semmelweis.hu (I.A.)

**Keywords:** bioinks, alginate, GelMA C, skin barrier, HaCaT, artificial epidermis, permeability, morphology, tissue engineering

## Abstract

In recent times, with the need for a reduction, refinement, and replacement of in vivo animal testing, there has been an increasing demand for the use of relevant in vitro human cell systems in drug development. There is also a great demand for the replacement of skin tissue in various wounds and burns. Furthermore, human skin cell-based in vitro systems can be used to investigate the side effects (toxicity and irritation) and tissue penetration of topical preparations. In this study, exploratory experiments were performed to produce artificial epidermis using two hydrogel scaffolds, alginate and GelMA C. The amount of keratinocytes added to the matrix (10–50–100 × 10^6^/mL) and the duration of tissue maturation (fresh, 1–3–4 weeks) were optimized in an extensive study. The behavior and structure of the two hydrogels were functionally and morphologically assessed. The permeability order for caffeine in the tested barriers was the following: alginate > GelMA C > cellulose acetate membrane > rat skin. It was concluded that GelMA C matrix provides a more favorable environment for cell survival and tissue differentiation (as demonstrated by histology and immunohistochemistry) than alginate. The 3-week incubation and 50 × 10^6^/mL cell number proved to be the most beneficial in the given system. This study provides data for the first time on the multifactorial optimization of two potential skin substitutes for tissue manufacturing. In order to use these results in tissue engineering, the fabricated artificial epidermis preparations must also be optimized for biocompatibility and from physical and mechanical point of views.

## 1. Introduction

The skin is our largest organ, which separates our body from the environment and protects us from physical, chemical, and biological/microbiological influences. It ensures the body’s homeostasis and is also an important platform for the application of medication and cosmetic treatments. Its anatomical structure is provided by three main layers, the epidermis, the dermis, and the subcutis. The uppermost layer of the epidermis is the stratum corneum, which consists of dead corneocytes located close to each other and embedded in a lipid matrix. The layer below is the viable epidermis, in which, in addition to keratinocytes as the main cell type, there are also melanocytes, Langerhans, and Merkel cells, which have an immunological role. This layer plays the most important role in the barrier function of the skin. The second layer, the dermis, contains capillary vessels (endothelial cells), hair follicles, sweat glands, and ducts, as well as sebaceous glands, neuronal elements, and fibroblasts [1]. The structure of this layer is looser and plays a significant role in the elasticity and mechanical properties of the skin, which is ensured by the extracellular matrix elements such as collagens, fibronectin, elastin, and fibrillins. The third layer is the subcutis, or hypodermis, the thickness of which varies greatly in different parts of the body and in the case of different individuals. The main cell type in this layer is the adipocytes, which play an important role in thermoregulation. Researchers are currently working on the development of several skin models, depending on the purpose for which the given artificial skin substitute is to be used. A possible and very important area of indication of application of skin equivalents is skin replacement and regeneration, which would be a crucial option in the case of acute and chronic wounds and burns [2,3]. This requires the production of full-thickness, vascularized skin tissue [4]. However, less complex in vitro skin substitutes can be used for other purposes as well, for example, to test the main effect or side effect (irritation, toxicity) of topical pharmaceutical preparations or cosmetics. For this purpose, many companies have developed models of different complexity, such as human reconstructed epidermis and full-thickness skin (Epiderm and EpiDermFT from MatTek Corporation, Ashland, MA, USA; EpiSkin from L’Oréal, Lyon, France; SkinEthic from SkinEthic, Lyon, France; EpiCs from CellSystems, Troisdorf, Germany; Straticell from Straticell, Les Isnes, Belgium; GraftSkin from Apligraf Organogenesis, La Jolla, Ca, USA; VitroLife-Skin, Kyoto, Japan).

In the present study, we developed and optimized a relatively low-complexity skin substitute for permeability studies, in which the emphasis was placed on the establishment of cell–cell interactions and barrier formation and the appropriate initial parameters and components were determined. The current series of experiments can serve as a starting point for the selection of proper systems and cell–bioink compositions. In the experimental system presented here, we show a comparative evaluation of two hydrogels, alginate and GelMA C, which, combined with human keratinocytes (HaCaTs), are able to form an epidermal barrier of varying degrees of efficiency. By examining the permeability, histological structure, and intercellular connections of the created skin models, a recommendation for the development of future artificial skin equivalents was formulated, which is given at the end of this article. The novelty of this study was that different conditions of tissue engineering were analyzed in a systematic way to assist the later steps of artificial epidermal tissue manufacturing.

Sodium alginate-based hydrogels are one of the most studied hydrogel systems in the field of 3D bioprinting due to their good biocompatibility, low cost, and excellent printability and versatility [5]. They have structural similarity to the native extracellular matrix (ECM) due to their similarity to the glycosaminoglycans (GAGs) in the human body [6]. The skin’s extracellular matrix is composed of a variety of biological macromolecules that have both structural and functional roles, including different types of collagens, glycosaminoglycans, proteoglycans, fibrillins, laminins, and integrins [7]. The fast gelation and shear-thinning properties of the alginate account for its good printing fidelity properties [8,9]. Despite all these benefits, alginate does not possess cell-activating sites to improve cell viability and has poor degradation. One of the commonly used polymers in combination with alginate is gelatin, which is a thermo-responsive biopolymer derivative from collagen. Gelatin is a biocompatible, bioresorbable biopolymer rich in arginine, glycine, and aspartic acid (Arg-Gly-Asp/RGD) motifs that help in cell attachment. Although gelatin offers cell adhesion motifs, it does not provide any other bioactive cues. Also, it has higher enzymatic degradation rates and low mechanical stability due to its higher solubility in the physiological environment. This can be improved by incorporating natural and synthetic polymers and inorganic materials to increase the stability of the system [10,11].

Gelatin methacryloyl (GelMA) has attracted the widespread interest of researchers because of its excellent biocompatibility, biodegradability, and moldability [12,13,14,15]. Various structures have been constructed from GelMA hydrogel, including 3D scaffold, injectable gel, bio-printed scaffold, and electrospun fibrous membrane, via precise fabrication methods such as light-induced crosslinking, extrusion 3D printing, electrospinning, or microfluidics. Due to its unique characteristics and simple preparation, GelMA hydrogel demonstrates superior performance and promising potential in a broad range of biomedical applications involving wound healing, drug delivery, biosensing, and tissue regeneration [15,16].

HaCaT cells are a spontaneously immortalized, human keratinocyte cell line that has been widely used for studies of skin biology and differentiation [17]. This cell line was used in the current study to generate epidermal barrier formation in hydrogels.

## 2. Materials and Methods

### 2.1. Bioinks (Hydrogels)

In the current study, two hydrogels were compared. The first hydrogel contained 3% of alginate (Merck-Sigma-Aldrich, Darmstadt, Germany) and 1% of gelatine (Merck-Sigma-Aldrich, Darmstadt, Germany). Typical alginate concentrations for extrusion-based bioprinting usually range between 2% and 4% (*w*/*v*), though this can vary depending on the specific application requirements (e.g., cell type, desired mechanical properties, printer settings). Concentrations around 2–4% (*w*/*v*) often provide a good balance between printability (viscosity) and biocompatibility. Slightly higher concentrations (e.g., up to 5% *w*/*v*) can improve structural fidelity but may reduce cell viability, while lower concentrations (1–2% *w*/*v*) can improve cell viability at the expense of print resolution [18,19,20]. HaCaT cells were loaded into the hydrogel in the proper concentrations (10 M/mL, 50 M/mL, and 100 M/mL). Discs with 6 mm diameter and 1.77 mm thickness were created by pipetting and stabilized by crosslinking with 200 mM CaCl_2_ solution immediately to prevent dehydration. Following a two-minute incubation period, the structures were washed twice with phosphate-buffered saline (PBS, 137 mM NaCl, 2.7 mM KCl, 10 mM Na_2_HPO_4_, 1.8 mM KH_2_PO_4_) and stored in PBS until the permeability experiment.

The second hydrogel GelMa C (CellInk, Gothenburg, Sweden) contained gelatin methacrylate (45–55% [21]) and nanofibrillated cellulose. Gelatin methacrylate already provides cells with biological cues, thanks to the natural adhesion motifs retained from gelatin, but supplementing it with nanofibrillated cellulose further enhances its rheological and mechanical properties, making it more suitable for skin bioprinting. Specifically, the nanofibrils increase viscosity and shear-thinning behavior, which improves print fidelity and structural integrity once printed. At the same time, their highly porous, fibrous network can aid in water retention and nutrient transport, thus supporting cell viability and proliferation. Additionally, the reinforcing effect of cellulose fibers helps the bioink better mimic the native skin extracellular matrix by offering a more robust, yet still biocompatible, environment for keratinocytes, fibroblasts, and other relevant cell types to attach, grow, and differentiate. It was loaded with HaCaT cells in the proper concentrations (10 M/mL, 50 M/mL, 100 M/mL). Discs with 6 mm diameter and 1.77 mm thickness were created by pipetting, stabilized by photo-crosslinking using UV light for 2 min, and stored in PBS until the permeability experiment.

### 2.2. Rheological Investigation of Hydrogels

Rheological measurements of both hydrogels prior to crosslinking were performed using a Kinexus Pro Rheometer (Malvern Instruments Ltd, Malvern, Worcestershire, UK), with data acquisition managed through rSpace for Kinexus Pro 1.3 software. PU20 SC0202 SS plate geometry was employed, maintaining a 0.1 mm gap for sample placement. Rotational tests at a controlled shear rate (0.1–100 s^−1^) were conducted at 25 °C, with temperature regulation ensured by the instrument’s Peltier system (±0.1 °C). During each measurement, a cylindrical stainless steel cover was placed over the sample to create a closed, saturated environment and prevent evaporation [18].

### 2.3. Scanning Electron Microscopy

After crosslinking, the alginate and GelMA C bioinks were frozen in liquid nitrogen and freeze-dried for scanning electron microscopy (SEM) analysis. Because a tabletop SEM (Hitachi TM4000Plus II) was employed, which allows imaging without additional conductive coatings, conductive sputter coating was unnecessary. The images were acquired at a 15 keV acceleration voltage in secondary electron mode (SE) and with an 11 mm working distance.

### 2.4. Cells and Culturing

The HaCaT cell line was obtained from Cell Lines Service (CLS, Heidelberg, Germany). Cells were cultured in Dulbecco’s Modified Eagle’s Medium (DMEM) high-glucose medium (Biosera, Cholet, France) supplemented with 10 *v*/*v*% heat-inactivated fetal bovine serum (FBS; Biosera, Cholet, France), 4 mM L-glutamine (Biosera, Cholet, France), and 1% penicillin–streptomycin (Biosera, Cholet, France) at 37 °C in a humidified atmosphere with 5% CO_2_. Medium exchange or subculturing occurred every 2nd or 3rd day. The HaCaT cells used for the experiments were between passage numbers 20 and 60.

Cell counts were determined using the trypan blue dye exclusion method with a Bürker chamber. Initially, cells were seeded at densities of 5 × 10^5^ cells/12 mL/T75 flasks (Sarstedt, Nümbrecht, Germany). Upon reaching the desired cell number, cells were suspended in the bioinks as described above. We loaded 50 µL of cell-containing bioink into an aluminum frame to form a disc (see Section 2.1). The alginate scaffolds were crosslinked with a 200 mM CaCl_2_ solution for 2 min, while the GelMA C discs were stabilized with UV exposure for 2 min. Samples were washed with phosphate-buffered saline and cultured for 1, 2, 3 or 4, weeks in DMEM (refreshed every 2nd or 3rd day).

### 2.5. In Vitro Viability Study on HaCaT Cells (Sulforhodamine B Assay-SRB)

Cell viability after exposure to the diluted hydrogels was determined by a colorimetric SRB assay. HaCaT cells (3000 cells/well) were seeded into 96-well plates, incubated at 37 °C overnight to let them attach, and treated with diluted alginate and GelMa C hydrogels. The hydrogels (alginate and GelMa C) were preheated to 37 °C and diluted at ratios of 1:1 and 1:8 in DMEM supplemented with 10% FBS before being added to the cells. Following 72 h of incubation, the medium was discarded, the cells were washed twice with phosphate-buffered saline solution (Gibco, Grand Island, NJ, USA; pH 7.4), and the cellular proteins were fixed by adding 10% trichloroacetic acid (TCA; Sigma–Aldrich, Steinheim, Germany) and incubating for 1h at 4 °C. After removing the TCA, the wells were washed in tap water, air-dried, and stained with 0.4% SRB dye solution (Sigma–Aldrich, Steinheim, Germany) in 1% glacial acetic acid for 15 min at room temperature. Unbound SRB was removed by washing with 1% acetic acid before air drying. The bound stain was solubilized with 10 mM Tris-HCl buffer (pH 8), and the optical densities were read on an automated spectrophotometric plate reader at a single wavelength of 570 nm [22,23,24].

### 2.6. Histology and Immunohistochemistry

For the detailed visual analysis of tissue formation in cell-containing bioinks, classical histological methods were chosen. Modern histological software enables more precise measurements, facilitating the quantification of observations. One of the most common staining methods, which utilizes hematoxylin and eosin, was applied. For the histological analysis 4 µm thick hematoxylin–eosin (HE)-stained slides were produced from the cell-containing hydrogel discs by the standard histological procedure from formalin-fixed paraffin-embedded blocks. The slides were scanned by a 3DHistech Panoramic 250 Flash III scanner (3DHISTECH Ltd., Budapest, Hungary).

From the same paraffin blocks, sections were also cut for immunohistochemistry. Antibodies for E-cadherin (Dako, M361201-2, Agilent Technologies, Novo-Lab, Törökbálint, Hungary) and Claudin 4 (Invitrogen; 32-9400, Thermo-Fischer Scientific, Budapest, Hungary) were used with an ultraView Universal DAB Detection Kit (5269806001) and stained by a Roche-Ultra plus stainer, according to the manufacturer’s recommendation.

### 2.7. Caffeine Cream Formulation

Caffeine cream was used as a hydrophilic model formulation to test the barrier function of the artificial skin models. The caffeine cream was prepared with the following composition [25]): caffeine (Thermo Fisher Scientific, Budapest, Hungary) active ingredient, 2%; polyoxyethylene sorbitan monostearate (Polysorbate 60) (Hungaropharma, Budapest, Hungary), hydrophilic emulsifying agent, 1.8%; alcohol cetylstearylicus (Molar Chemicals, Halásztelek, Hungary), lipophilic emulsifying agent, 5.5%; paraffinum liquidum (Hungaropharma, Budapest, Hungary), lipophilic base, 7.7%; paraffinum album (Hungaropharma, Budapest, Hungary), lipophilic base, 12.0%; propylene glycol (Hungaropharma, Budapest, Hungary), antimicrobial agent preservative, stabilizer, 14.6%; and purified water, hydrophilic phase, 56.4%. The cream was prepared ex tempore, as was described earlier by our group [26,27].

### 2.8. In Vitro Permeation Study in Skin-on-a-Chip Microfluidic Device

**Figure 1:**
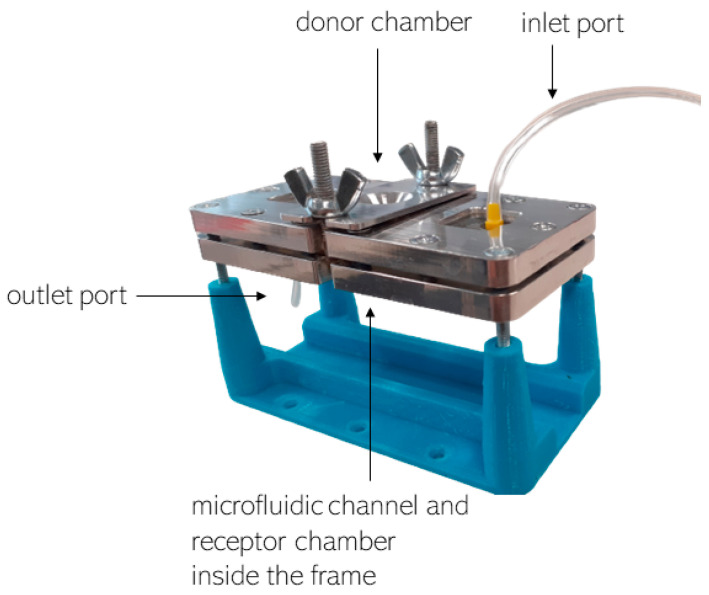
Skin-on-a-chip microfluidic diffusion chamber.

A skin-on-a-chip microfluidic diffusion chamber (Figure 1) was used for the permeation studies, as was described earlier [25,26,28,29]. It contains two compartments (donor and acceptor), and the barrier model (the skin or skin substitute) was placed between them. The following barrier models were tested: (1) blank and cell-containing alginate scaffolds, (2) blank and cell-containing GelMA C scaffolds, (3) cellulose acetate membrane (pore size: 0.45 µm, Sartorius AG, Göttingen, Germany) soaked in isopropyl myristate for 30 min prior to the experiment, and (4) rat skin after 10 rounds of tape stripping (male Wistar rats weighing 572–615 g, ToxiCoop Zrt., Budapest, Hungary). The diffusion surface was 0.283 cm^2^, which separated the cream-containing donor chamber and the receptor chamber, filled with peripheral perfusion fluid (PPF, consists of 147 mM NaCl, 4 mM KCl, and 2.3 mM CaCl_2_, all substances acquired from Sigma-Hungary Kft., Budapest, Hungary). The PPF flow was continuous in the receptor compartment with a flow rate of 4 μL/min, ensured by a syringe pump (NE-1000, New Era, Farmingdale, NY, USA). The samples were collected every 30 min for 5 h. Caffeine concentrations of the perfused physiological fluid samples were determined with an UV-VIS spectrophotometer (NanoDrop™ 2000, Thermo Scientific, Budapest, Hungary) immediately after each sample collection. The absorption maximum of caffeine was detected at 272 nm.

### 2.9. Statistics

The cumulative mass values were averaged, and means and standard error of mean (SE) were calculated and presented in graphics. Area under the curve (AUC) values were calculated using the trapezoidal method. For statistical comparison, one-way ANOVA followed by Tukey’s post hoc test was applied using OriginPro 2022 (OriginLab Corporation, Northampton, MA, USA) software. A *p*-value < 0.05 was considered statistically significant.

## 3. Results

### 3.1. Rheological Comparison of Hydrogels

**Figure 2:**
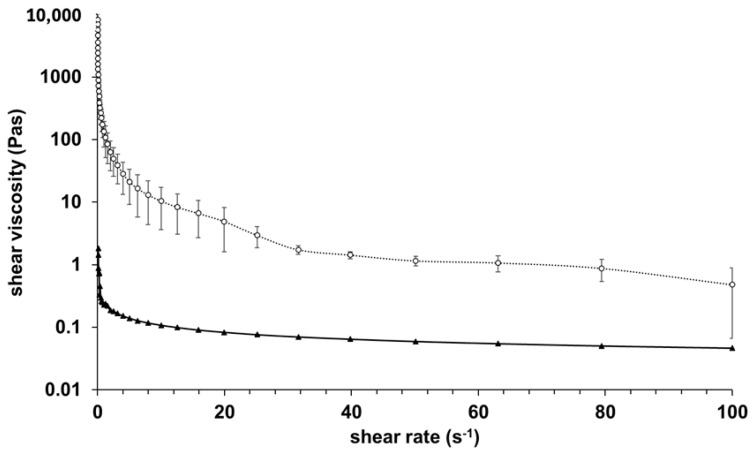
Viscosity curves of alginate (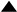) and GelMA C (**O**) gel samples before crosslinking with a controlled shear rate (0.1–100 1/s) at 25 °C.

The results of rheological investigations of alginate and GelMA C hydrogels are presented in Figure 2.

The samples were very different in their viscosity, with GelMA C significantly more viscous at 25 °C; however, both showed shear-thinning behavior. This behavior can be characteristic of the entanglement of polymer molecules with an increasing shear rate.

### 3.2. Morphological Comparison of Hydrogels

To better understand the topography and internal structures of hydrogels, the corresponding cross-sectional and superficial microstructures of acellular discs were examined by scanning electron microscopy.

**Figure 3:**
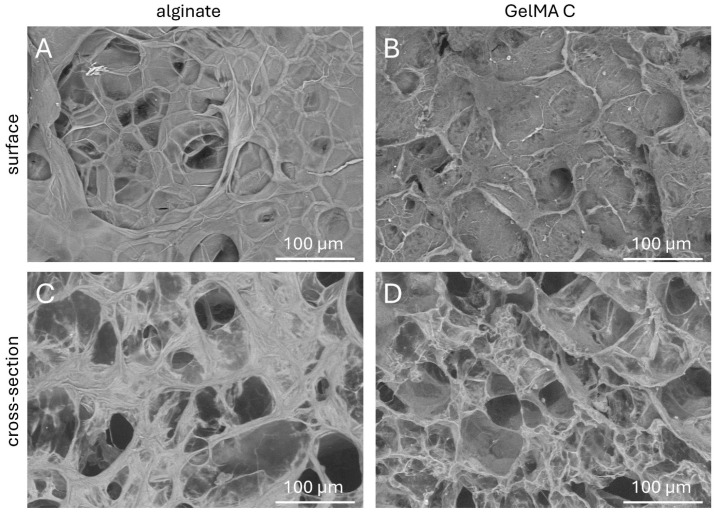
Scanning electron microscopic images of the surface and cross-sectional view of (**A**,**C**) alginate and (**B**,**D**) GelMa C scaffolds after crosslinking.

As is shown in Figure 3C,D, the cross-sectional microstructures of the GelMA C hydrogel visually exhibited a loose, honeycomb-like morphology with different pore sizes, most probably due to the variable degree of substitution of gelatine methacrylate of the hydrogel during photo-crosslinking. Meanwhile, alginate showed a more compact structure, though also a wide variety of pore sizes. The superficial images presented a relatively smooth surface for alginate and more irregular characteristics for GelMA C (Figure 3A,B).

In our study, acellular gel discs were subjected to SEM imaging following freezing and a freeze-drying process. During this process, many ice crystals form, which then grow until they bump into neighboring ice crystals and can no longer grow any further. The base material of the gel does not become part of the crystals, but is displaced by them and then concentrates between the crystals. The ice crystals are then removed by freeze-drying, leaving a pore at the location of a crystal. The hydrated gels have different internal structures, as was reported earlier [30]. Vacuum-based techniques, such as scanning electron microscopy (SEM), are still being commonly used to measure pore sizes in hydrogels, which is often not representative of the actual pore size in hydrated conditions [31,32].

**Figure 4:**
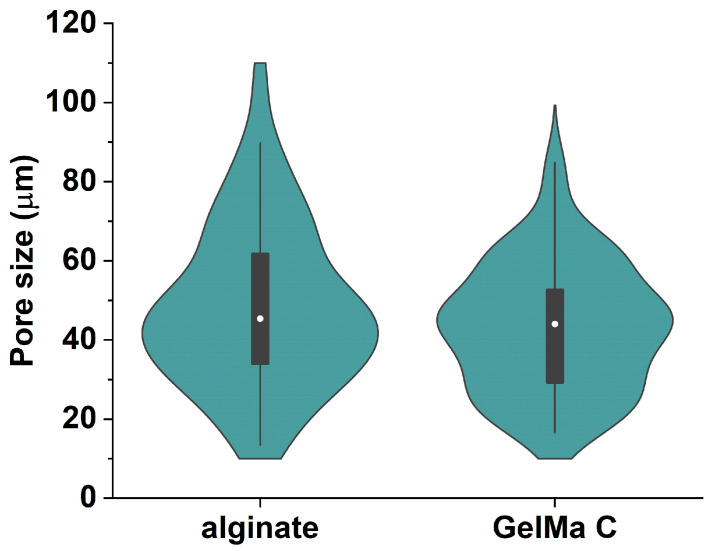
The pore size distribution in the internal structure of alginate- and GelMa C-based hydrogel matrices following freezing and freeze-drying process. No statistically significant difference was found between the two gels.

The SEM images were subjected to image analysis to acquire more information about the pore size and distribution frequency of the cavities within the internal structure. The results are presented in Figure 4 in a violin plot. The average pore sizes were 48.22 ± 2.89 μm in alginate scaffolds and 43.08 ± 2.27 μm in GelMaC.

### 3.3. Viability Study on Keratinocytes

**Figure 5:**
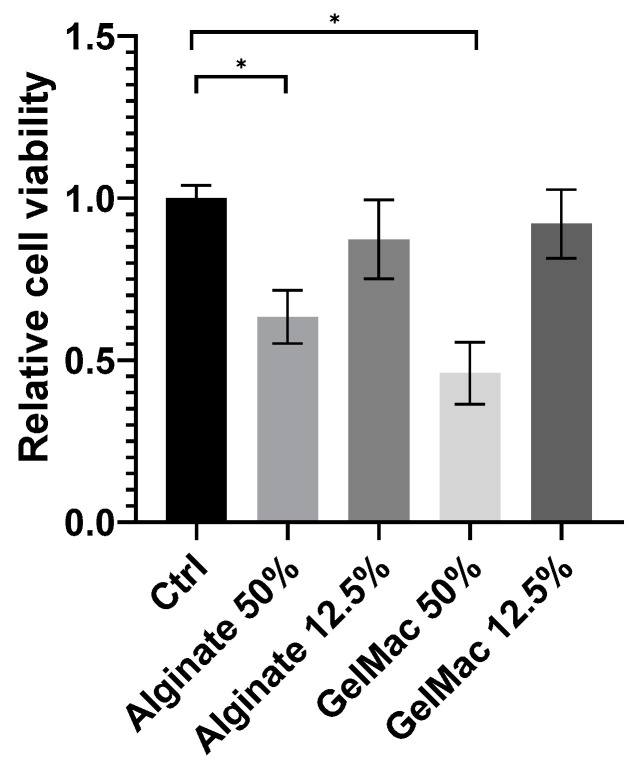
Effect of diluted hydrogels on cell viability, determined using SRB assay and HaCaT cells (means ± SD), *: *p* < 0.05, n = 2 × 5.

Based on the results of the SRB assay (Figure 5) on alginate and GelMa C hydrogels (before chemical or physical stabilization by crosslinking), they expressed a concentration-dependent and statistically significant inhibitory effect on the viability of keratinocytes. However, it cannot be judged by this assay, whether this is a cytotoxic or cytostatic effect. Several authors investigated the different variations and formulations of alginate and GelMa scaffolds for biocompatibility [33,34,35] under various conditions (diverse incubation times and cell numbers were applied). The majority of the authors tested the scaffolds after crosslinking and reported a remarkable effect of the hydrogels on cell viability. As was demonstrated, by tailoring the parameters of the polymers (e.g., encapsulation, chemical conjugation, nano-formulation), the toxic effect can be weakened, providing a better chance of application in tissue engineering [36].

In this stage of the study, we performed no detailed evaluation of the fixed, cell-containing matrices. The viability assay we used provided only preliminary information about the possible cytotoxic effects of the gels and about their relative toxicity compared to each other on HaCaT keratinocytes. To analyze these effects further, more relevant and more sophisticated assays are needed. Counting the cells in the fixed gels is an option, to characterize the survival of the cells in the matrix. A separate study is planned for an extensive evaluation of the cell-containing gels.

### 3.4. Penetration Studies

In the current study, only one model formulation was tested. We used the 2% (*w*/*v*) caffeine in o/v emulsion cream. Caffeine is widely applied in cosmetic products due to its high biological activity and ability to penetrate the skin barrier [37,38,39]. This alkaloid is a hydrophilic model substance and has no ideal properties for transcellular skin permeation due to its physicochemical characteristic (M.W. 194.08, log Ko/w: −0.07) [40]. However, it was reported earlier that 51.0% of topical caffeine penetrates the epidermis via the follicular pathway, and 63.9% of it reaches the dermis by the transappendageal route. In intact skin, topical caffeine was detectable in the systemic circulation after 5 min, while in case of the closed follicle technique (CFT), the molecule appeared in the plasma just after 15 min. These observations confirm that this molecule greatly uses the transappendageal penetration routes during skin absorption. In our case, the compound should cross the cell-containing matrices. Caffeine can penetrate partially transcellularly through the cellular aggregates, but mainly through the hydrogel matrices. Thus, the properties of the matrices themselves can be determining in the kinetics of this transport.

#### 3.4.1. Cell Number Optimization

**Figure 6:**
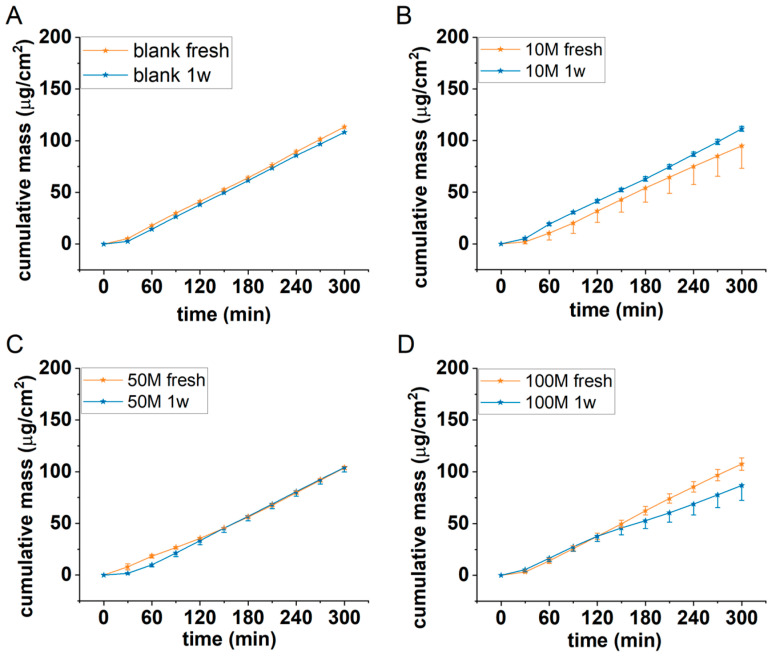
Cumulative mass–time profiles of caffeine permeation through (**A**) acellular, and (**B**) 10^7^/mL, (**C**) 5 × 10^7^/mL, and (**D**) 10^8^/mL HaCaT cell-containing alginate discs at room temperature (RT). N = 3. 1w = one-week maturation.

In the first series of experiments, the optimum cell concentration was obtained in alginate scaffolds using HaCaT cells. Acellular (blank) and 10–50 and 100 × 10^6^/mL cell-containing alginate discs were tested for permeability using 2% caffeine cream as a model formulation. The experiments were performed in a microfluidic diffusion chamber in freshly prepared discs and in discs after one week of cell maturation. The results are presented in Figure 6.

Based on our results, it was concluded that the barrier function of the cell-containing scaffolds was the strongest in the case of a 50 M/mL cell concentration and in the case of 1 week of maturation. The permeability levels of the scaffolds with lower (10 M/mL) and higher (100 M/mL) cell numbers were higher than and comparable with the blank alginate scaffolds, respectively.

#### 3.4.2. Incubation Time Selection

**Figure 7:**
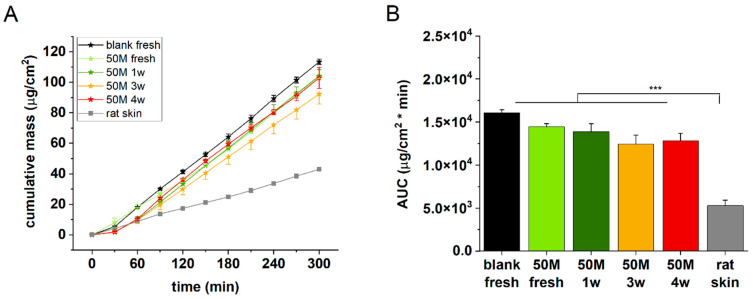
(**A**) Cumulative mass–time profiles of caffeine permeation through alginate scaffolds and abdominal rat skins. (**B**) Area under the cumulative mass–time curves. n = 3, ***: *p* < 0.001.

To create better cell–cell interactions and therefore a more relevant barrier function in the artificial epidermal tissue, in the next experiments, extended incubation times were tested. Freshly prepared and 1–3- and 4-week-matured discs containing 50 M/mL HaCaT cells were examined for permeability in comparison with acellular alginate and ex vivo rat skin. The best barrier function, as expected, was detected for the natural excised skin (Figure 7A,B), followed by the 3- and 4-week-incubated cell-containing alginates. Based on these findings, the next studies were performed after 3 weeks of maturation with 50 M/mL cell-containing alginate discs.

### 3.5. Histological and Immunohistochemical Analysis of Cell-Containing Scaffolds

**Figure 8:**
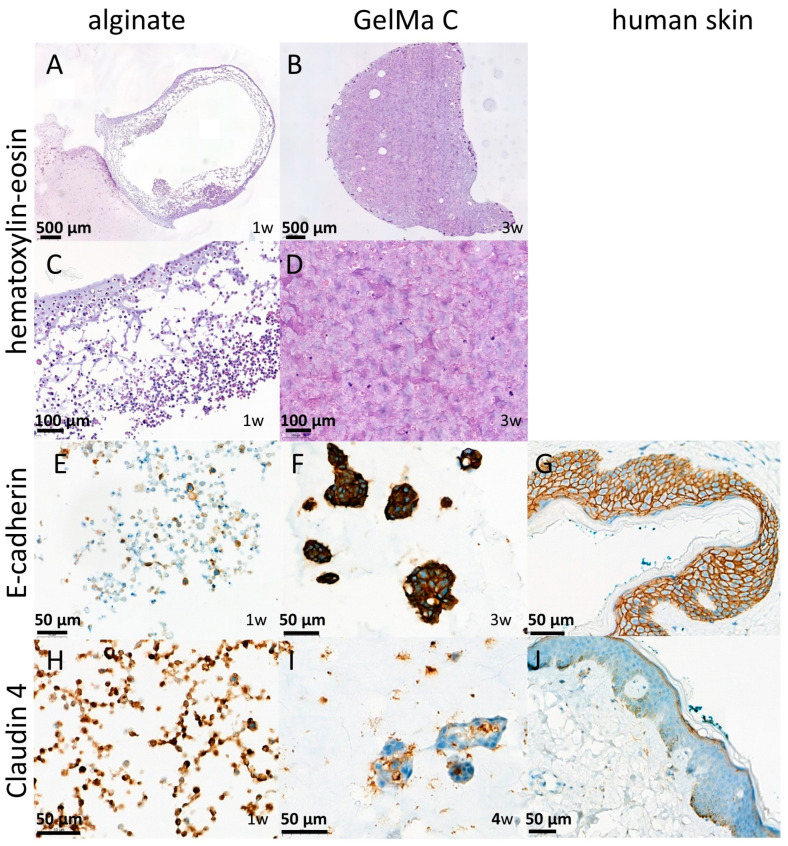
Histological and immunohistochemical characterization of HaCaT cells containing alginate (**A**,**C**,**E**,**H**) and GelMA C (**B**,**D**,**F**,**I**) hydrogel scaffolds. Classical histology (HE staining) (low-power images (**A**,**B**); high-power images (**C**,**D**)) and E-cadherin (**E**–**G**) and Claudin 4 (**H**–**J**) immunohistochemistry were applied. For reference and validation of the immunohistochemistry antibodies, human skin samples were used in parallel (**G**,**I**).

To confirm the results of our functional investigation, the alginate and GelMA C scaffolds were also studied for their morphology using classical histology (HE staining) [41], and for evaluation of the cell–cell interactions and level of tissue-like formation, E-cadherin and Claudin 4 immunohistochemistry was applied on the sections from the paraffin-embedded discs. E-cadherin is a transmembrane glycoprotein which connects epithelial cells together at adherens junctions, while Claudin 4, a transmembrane protein, is located at the tight junctions between neighboring cells. Both proteins are the markers of cell–cell connections. As shown in Figure 8 panels A and B, the cells were able to survive 1 week of incubation only in the marginal zone of the alginate discs, indicating that insufficient oxygen and nutrients were available in the core, while in GelMA C scaffolds, a better cell distribution was observed. These findings can be explained by the different matrix structures of the two hydrogels, the higher viscosity of the GelMA C, and the better adherence and biocompatibility of this bioink.

Figure 8 panels C and D show that E-cadherin, a marker of cellular connections, was more prominent in the GelMA C scaffold, where the keratinocytes were able to form aggregates and functional units within the bioink; this was contrary to alginate, where the cells remained separate without any visible connections.

In the case of Claudin 4, the junctional connections were visualized. In Figure 8 panels F and G, remarkable adherens junctional connections can be seen within the alginate matrix, while in the cell aggregates within the GelMA C bioink, very strong Claudin 4 expression was detected, indicating a good matrix environment for tissue formation and cellular connectivity for HaCaT cells.

The values calculated for the permeability of this artificial epidermis include the processes of penetration through the HaCaT cells and penetration through the matrices. At the current stage of this research, it is not possible to judge the proportion of transcellular penetration and the extracellular penetration components within the scaffold. However, it can be supposed that with the progress of the cell–cell interactions and improving complexity of tissue formation (in this case, not monolayer but 3D tissue formation), the barrier function becomes stronger and consequently the permeability will be reduced.

### 3.6. Comparison of Alginate and GelMa C Scaffolds

**Figure 9:**
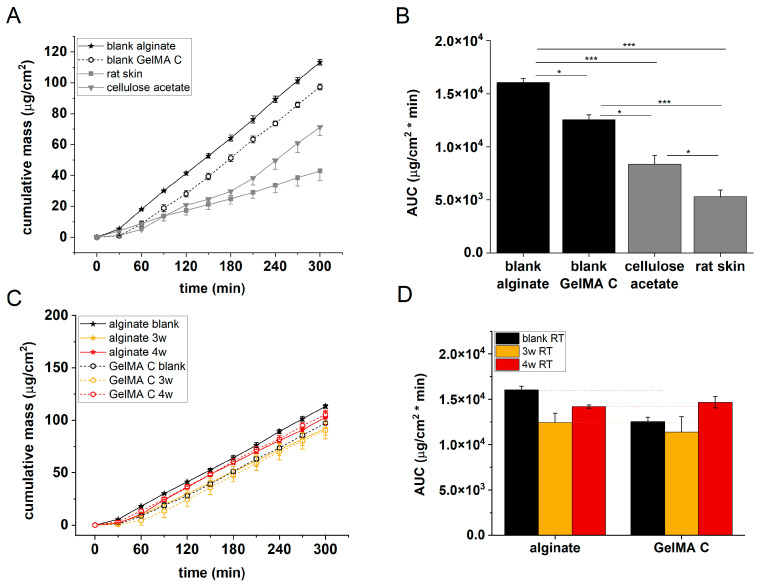
(**A**,**C**) Cumulative mass–time profiles of caffeine permeation through cellulose acetate membrane, rat skin, alginate, and GelMA C scaffolds. (**B**,**D**) Area under the cumulative mass–time curves. n = 3, *: *p* < 0.05, ***: *p* < 0.001. 3w: three weeks of maturation, 4w: four weeks of maturation, RT: room temperature.

The permeability of acellular alginate and GelMA C scaffolds, cellulose acetate membrane, and excised rat skins was tested in microfluidic diffusion chambers. As can be seen in Figure 9, the penetration order of caffeine was the following: alginate > GelMA C > cellulose acetate membrane > rat skin. Concerning the histological and immunohistochemical features of the two scaffolds, different distributions and functional organizations of the cells can be observed within the hydrogels. In alginate, there was only a marginal presentation of the cells in the discs, most probably due to the insufficient diffusion of oxygen and nutrients through the scaffold, while in GelMA C hydrogel, the cells were able to survive in the central core of the discs and showed a homogenous distribution within the matrix.

Figure 9 panels C and D show that the barrier function of the cell-containing hydrogels was better after 3 weeks of incubation than after 4 weeks. This suggests that the viability of the cells in the scaffolds is limited, and that longer maturation does not mean better tissue formation.

## 4. Discussion

In this series of experiments, we focused on the development and evaluation of an artificial epidermis and bioink matrix composition, which may be suitable for future 3D bioprinting applications. Two bioinks, gelatin-containing alginate and GelMa C, were used, because although there are many types of bioinks on the market, such as collagen, fibrin, silk fibroin, and polyethylene glycol (PEG)-based materials, alginate and GelMA remain two of the most widely used hydrogel systems in bioprinting. The bioinks were combined with keratinocyte cells at varying concentrations. After different incubation periods, the resulting tissue-like constructs were analyzed both morphologically (using scanning electron microscopy, classical histology, and immunohistochemistry) and functionally (via permeability tests). The results indicate that, among the three incubation times tested, the 3-week period was the most favorable, with the barrier function being strongest at a concentration of 50 M/mL cells. This systematic, multi-parametric approach for the development of HaCaT cell-containing matrices represents a novelty in the tissue engineering of human cell-based artificial skin substitutes.

When comparing the two hydrogels, the GelMA C bioink clearly provided a more beneficial environment for cell maturation and development under the tested conditions than the alginate-based bioink. This suggests that GelMA C’s matrix properties offered better support for cell adhesion, intercellular communication, differentiation, and tissue formation in human keratinocytes. However, mechanical properties and the thermo-sensitivity of the artificial tissues were not assessed in this study.

Based on the results of permeation experiments, it can be concluded that both gels are unsuitable in the current form for skin tissue penetration testing. To reliably form a stratified epidermal layer using HaCaT cells, a suitable dermal support could be beneficial, for example, fibroblasts and extracellular matrix proteins in a supportive hydrogel or a basement-membrane-rich layer. Moreover, because in thick, dense hydrogels, diffusion of nutrients and oxygen can become limiting, reducing the thickness of the discs and transitioning to an air–liquid interface after initial cell proliferation could help in promoting the formation of an epidermal layer.

The gelation, rheological, mechanical, and biological aspects of a bioprinting solution are all equally important, playing a critical role in ensuring the successful printability, mechanical strength, and biofunctionality of scaffolds [42]. Gelation at room temperature is essential for potential tissue engineering applications, but it must be controllable to prevent issues during printing [43]. Rheological properties such as viscosity and shear thinning behavior are also crucial for the printability of a gel or solution. The bioink should have the appropriate viscosity to allow smooth flow with minimal obstruction and sufficient cohesion to prevent breaking during the printing process [44]. Shear thinning, a characteristic of non-Newtonian fluids, occurs when viscosity decreases under applied shear stress, and shear-thinning curves can be extremely helpful in comparing the shear rates of bioinks at similar viscosity levels, helping to select a bioink that extrudes well at lower pressures, ensuring better cell survival [45]. Temperature and external mechanical stimuli are the main controllable conditions in 3D bioprinting. The mechanical strength of scaffolds also impacts cell survival. Stiffer materials offer less space for cells to move and infiltrate, which could hinder their biofunctionality [46,47]. For example, a dense core in alginate discs might limit signal exchange and nutrient diffusion, reducing its regenerative potential [48,49]. Biocompatibility, biofunctionality, and biodegradability are crucial factors for bioinks used in 3D bioprinting [50]. To make meaningful recommendations for 3D bioprinting and tissue engineering of artificial human epidermis based on GelMA C hydrogel and human keratinocytes, further research is required.

## Data Availability

All data connected to this study are available from the authors on request.
